# Oral glucose tolerance test predicts increased carotid plaque burden in patients with acute coronary syndrome

**DOI:** 10.1371/journal.pone.0183839

**Published:** 2017-08-30

**Authors:** Thorarinn A. Bjarnason, Steinar O. Hafthorsson, Linda B. Kristinsdottir, Erna S. Oskarsdottir, Thor Aspelund, Sigurdur Sigurdsson, Vilmundur Gudnason, Karl Andersen

**Affiliations:** 1 Department of Medicine, Division of Cardiology, Landspitali the National University Hospital of Iceland, Reykjavik, Iceland; 2 University of Iceland, School of Health Sciences, Reykjavik, Iceland; 3 Icelandic Heart Association, Kopavogur, Iceland; Medical University Innsbruck, AUSTRIA

## Abstract

**Background:**

Type 2 diabetes and prediabetes are established risk factors for atherosclerosis. The aim of this study was to evaluate the atherosclerotic plaque burden in the carotid arteries of patients with acute coronary syndrome according to their glycemic status.

**Methods:**

Patients with acute coronary syndrome and no previous history of type 2 diabetes were consecutively included in the study. Glucose metabolism was evaluated with fasting glucose in plasma, HbA1c and a standard two-hour oral glucose tolerance test. Atherosclerotic plaque in the carotid arteries was evaluated with a standardized ultrasound examination where total plaque area was measured and patients classified as having no plaque or a significant plaque formation.

**Results:**

A total of 245 acute coronary syndrome patients (male 78%, 64 years (SD: 10.9)) were included. The proportion diagnosed with normal glucose metabolism, prediabetes and type 2 diabetes was 28.6%, 64.1% and 7.3%, respectively. A significant atherosclerotic plaque was found in 48.5%, 66.9% and 72.2% of patients with normal glucose metabolism, prediabetes and type 2 diabetes, respectively. An incremental increase in total plaque area was found from normal glucose metabolism to prediabetes (25.5%) and from normal glucose metabolism to type 2 diabetes (35.9%) (p = 0.04). When adjusted for conventional cardiovascular risk factors the OR of having significant atherosclerotic plaque in the carotid arteries was 2.17 (95% CI 1.15–4.15) for patients with newly diagnosed dysglycemia compared to patients with normal glucose metabolism. When additionally adjusted for the 2-hour plasma glucose after glucose loading (2hPG) the OR attenuated to 1.77 (95% CI 0.83–3.84).

**Conclusion:**

Newly detected dysglycemia is an independent predictor of significant atherosclerotic plaque in the carotid arteries with oral glucose tolerance test as a major determinant of carotid plaque burden in this group of individuals with acute coronary syndrome.

## Introduction

Type 2 diabetes mellitus (T2DM) and prediabetes are established risk factors for atherosclerosis and cardiovascular disease (CVD) and associated with increased mortality compared to those with normal glucose metabolism (NGM) [1]. Patients with acute coronary syndromes (ACS) have a high prevalence of undiagnosed T2DM and prediabetes [2, 3] at hospital admission. There is evidence that myocardial damage may already be present at the time of clinical diagnosis of T2DM indicating that the dysglycemia (T2DM or prediabetes) has remained undiagnosed for months or years [4]. Moreover, studies have indicated that newly diagnosed dysglycemia in patients with myocardial infarction is a major risk factor for future cardiovascular events [5, 6]. It therefore has been recommended to screen for metabolic derangements in patients admitted to hospital for ACS [7].

Atherosclerotic disease in the carotid arteries has been associated with coronary artery disease and is an indicator of an increased atherosclerotic burden [8]. Measuring total plaque area (TPA) in the carotid arteries is a noninvasive method to quantify atherosclerotic burden. Carotid plaque is also an independent predictor of CVD and improves risk stratification in addition to traditional risk factors for CVD [9, 10].

We hypothesized that patients with ACS have a high prevalence of atherosclerotic plaque in the carotid arteries and that ACS patients with newly diagnosed T2DM and prediabetes have increased atherosclerotic burden compared to ACS patients with NGM. Therefore, we set out to evaluate the atherosclerotic plaque burden in the carotid arteries of patients with ACS and related the plaque burden to newly detected metabolic derangements.

## Methods

### Study population

Patients admitted to the coronary care unit of Landspitali, the University Hospital of Iceland with the diagnosis of ACS were consecutively included in the study between June 2013 to October 2014. ACS was defined according to the joint European Society of Cardiology (ESC) and American College of Cardiology recommendations [11]. Patients with previously known T2DM, cognitive dysfunction, living in a nursing home or outside the catchment area of the hospital were excluded from the study.

Prior to any study related procedure informed written consent was obtained for each participant. The study protocol adhered to the principles laid out in the Declaration of Helsinki [12] and was approved by the Icelandic Bioethics Committee (VSN: 13-069-S1). Information on prespecified demographic, personal, medication and lifestyle data were obtained from patients during admission and from hospital records. Patients with previous history of hypertension or on blood pressure lowering medication were classified as having hypertension. Likewise, patients with previous history of hypercholesterolemia or on statins were classified as hypercholesterolemia. Patients with 1^st^ degree family members with coronary artery disease (CAD) were defined as having family history of CAD.

### Diagnosis of glucose metabolism

Glucose metabolism was evaluated after an overnight fast of at least ten hours with fasting plasma glucose (FPG), glycated hemoglobin (HbA1c) and a standard oral glucose tolerance test (OGTT). Two hours after ingesting a solution containing 75 g of glucose measurements of plasma glucose (2hPG) were made. Measurements of glucose metabolism were made during hospitalization, generally on third to fifth day after admission, and repeated at least three months later. All samples collected for venous plasma glucose measurements were centrifuged and analyzed immediately after blood collection. Glucose levels were determined using reagents, calibrators and Vitros 250/950 analyzers from Ortho Clinical Diagnostics, Rochester, USA and HbA1c levels were determined using reagents, calibrators and Cobas c311 analyzer from Roche, Mannheim, Germany.

The classification of glucose metabolism was based on the American Diabetes Association (ADA) criteria, where prediabetes was defined as HbA1c 5.7–6.4% (39–47 mmol/mol), FPG 5.6–6.9 mmol/l (100–125 mg/dL) or 2hPG 7.8–11.0 mmol/l (140–199 mg/dL) and T2DM as HbA1c ≥6.5% (48 mmol/mol), FPG ≥7.0 mmol/l (126 mg/dL) or 2hPG ≥11.1 mmol/l (200 mg/dL) [13]. Patients were classified as having T2DM if at least two measurements were above the cut-point for T2DM according to the ADA criteria while patients with one measurement above the cut-point of T2DM or at least one measurement above the cut-point for prediabetes were classified as having prediabetes.

### Carotid imaging

Three months after discharge an ultrasound imaging of the carotids was performed according to a standardized protocol [14]. A Toshiba Aplio 300 system with a two-dimensional 6.8 MHz linear array transducer was used for all ultrasound imaging by a trained sonographer. Standardized longitudinal B-mode images of the common carotid artery, bifurcation and internal carotid artery from the near and far wall in the left and right carotid arteries were examined. The lateral extent of the common carotid segment was defined relative to the tip of the flow divider, which is normally the most clearly defined anatomical reference in the proximity of the carotid bifurcation. The bifurcation and internal carotid segments were also defined by using the tip of the flow divider. The segments were defined as: the near wall and far wall of the arterial segment extending from 10 mm to 20 mm proximal to the tip of the flow divider into the common carotid artery (CCA); the near wall and far wall of the carotid bifurcation beginning at the tip of the flow divider and extending 10 mm proximal to the flow divider tip (BIF); and the near wall and far wall of the proximal 10 mm of the internal carotid artery (ICA). Images for the assessment of the carotid intima-media thickness (cIMT) were acquired from the predefined 10 mm segment of each CCA at defined interrogation angles using the Meijers arc [15] and standard images obtained from four angels at each site. The mean cIMT of the near and far walls were determined from a single image at each interrogation for both CCA and the mean cIMT values from these sites comprised the cIMT outcome parameter.

Atherosclerotic plaques in the carotid artery segments (BIF and ICA) extending 10 mm proximal and distal from the tip of the flow divider, were categorized as a significant or not. The most severe lesion per segment was assessed [16]. Patients with a least one, clear, reasonable easy to be visualized plaque with intima media thickness of at least twice the thickness of adjacent sites causing at least some diameter reduction of the vessel lumen were classified as having significant plaque. In addition, the Artery Measurements System software (v.2.02.) was used to assess quantitatively the plaque area of all visible plaques in the BIF and ICA segments. An atherosclerotic plaque was defined by an isolated thickening at least double the adjacent normal cIMT by visual assessment. The plaque boundaries were traced with a cursor on the computer screen and the area (mm^2^) for each plaque automatically computed by the program. The TPA was calculated by summing the area of all individual plaques. Acquisition and interpretations were made by trained sonographers [14].

### Severity of coronary atherosclerosis

All patients underwent coronary angiography during admission. Coronary arteries with lumen reduction of more than 70% were considered to have a significant stenosis and patients classified of having 0-, 1-, 2- or 3-vessel disease. Gensini score was applied to evaluate the coronary artery disease by the degree of lumen reduction and the importance of the lesion´s location [17].

### Statistical analysis

Categorical variables were presented as percentage. Normally distributed continuous variables were presented as means (standard deviation, SD), otherwise as median with interquartile range (IQR). Between group comparison of NGM, prediabetes and T2DM was made using the chi-square test and analysis of variance or Kruskal Wallis test for categorical and continuous variables, respectively. The odds ratio (OR) of having significant plaque in the carotid arteries was estimated with a multivariable logistic regression model. The predictors used were HbA1c, FPG and 2hPG. The association between TPA and risk factors for atherosclerosis was evaluated with general linear regression models, both unadjusted and adjusted for glucometabolic factors and conventional atherosclerotic risk factors: age, gender, hypertension, hypercholesterolemia, smoking status and BMI. The level of statistical significance was set at *p*<0.05. All statistical analyses were performed using the R software version 3.2.2. [18]

## Results

A total of 435 patients admitted to the coronary care unit with the diagnosis of ACS were considered for inclusion in the study. Among these, 90 were discharged before the OGTT could be performed, 60 refused participation and two died shortly after admission. Therefore, 283 patients were included in the study shortly after admission. Before the follow-up visit at 3 months, 21 patients were lost to follow-up, 13 patients withdrew consent, three died and one had a stroke. The remaining 245 patients comprised the study population.

The baseline characteristics of the study population are presented in Table 1. The mean age was 64.0 (SD 10.9) years, 78.0% were male and all participants in the study were Caucasian of European origin. A total of 28.6%, 64.1% and 7.3% were diagnosed with NGM, prediabetes and T2DM, respectively. The mean TPA and cIMT in the study population was 103.3 mm^2^ (SD 64.7) and 0.90 mm (SD 0.15), respectively.

**Table 1:** Baseline characteristics.

	(n = 245)
Age, years (SD)	64.0 (10.9)
Gender	
- Male	191 (78%)
- Female	54 (22%)
Glucose metabolism	
- At admission:	
HbA1c, % (SD)	5.4 (0.5)
FPG, mmol/l (SD)	5.3 (0.9)
2hPG, mmol/l (SD)	7.8 (2.6)
- At follow-up:	
HbA1c, % (SD)	5.5 (0.4)
FPG, mmol/l (SD)	5.5 (0.8)
2hPG, mmol/l (SD)	6.7 (2.6)
hs-CRP, mg/L (IQR)	1.1 (0.6, 2.3)
Insulin, mU/L (SD)	12.8 (8.6)
HOMA-IR (SD)	3.2 (2.4)
TPA, mm^2^ (SD)	103.3 (64.7)
Grey-scale median (SD)	37 (10)
Mean cIMT, mm (SD)	0.90 (0.15)
Carotid plaque	
- No plaque	93 (38.0%)
- Significant plaque	152 (62.0%)
BMI, kg/m^2^ (SD)	28.7 (4.2)
WHR (SD)	0.98 (0.07)
Smoking status:	
- Never	32.2%
- Previous	44.1%
- Current	23.7%
FH of CAD^a^	65.7%
Hypercholesterolemia	46.1%
Hypertension	56.7%
Previous CAD	33.5%
SBP, mmHg (SD)	139 (22)
DBP, mmHg (SD)	77 (13)
ACS	
- UAP	30.2%
- NSTEMI	40.4%
- STEMI	29.4%
CAD ^b^	
- 0-vessel	7.8%
- 1-vessel	39.8%
- 2-vessel	28.7%
- 3-vessel	23.7%
Gensini score (SD)	40.4 (34.6)

SD: standard deviation, FPG: fasting plasma glucose, 2hPG: 2-hour plasma glucose, hs-CRP: high-sensitivity C-reactive protein, IQR: interquartile range, HOMA-IR: homeostatic model assessment insulin resistance, TPA: total plaque area, cIMT: carotid intima-media thickness, BMI: body mass index, WHR: waist to hip ratio, FH: family history, CAD: coronary artery disease, SBP: systolic blood pressure, DBP: diastolic blood pressure, ACS: acute coronary syndrome, UAP: unstable angina pectoris, NSTEMI: non-ST elevation myocardial infarct, STEMI: ST elevation myocardial infarct

^a^CAD with 1^st^ degree family member

^b^≥70% of lumen reduction

At least one atherosclerotic plaque was detected in 48.5%, 66.9% and 72.2% of patients with NGM, prediabetes and T2DM, respectively (Table 2). A significant difference in TPA was found between patients diagnosed with NGM (86.8 mm^2^ (SD 57.2)) prediabetes (108.9 mm^2^ (SD 66.1)) and T2DM (118.0 mm^2^ (SD 70.7)) (*p*<0.04). The incremental increase in TPA was 25.5% and 35.9% among patients diagnosed with prediabetes and T2DM, respectively, compared to patients with NGM. A significant difference in BMI, waist-to-hip ratio, smoking status, high-sensitivity C-reactive protein (hS-CRP), insulin resistance (HOMA-IR), number of diseased coronary arteries and Gensini score was found between different glucose metabolism categories (Table 2). Increased TPA was detected among patients with impaired glucose tolerance (IGT), either alone or in combination with impaired fasting glucose (IFG), compared with patients with isolated IFG (Table 3). No difference was detected in cIMT between patients with different glucose metabolism categories while an association with cIMT was found between different age categories (per 5 years) and gender, 0.030 mm (SE; 0.004, p<0.01) and 0.057 mm (SE; 0.022, p = 0.01), respectively.

**Table 2:** Pertinent characteristics of patients divided according to their glucose metabolism.

	NGM (n = 70)	Prediabetes (n = 157)	DM (n = 18)	p-value
Age, years (SD)	64.0 (12.0)	63.9 (10.2)	65.0 (12.5)	0.92
Gender				0.54
- Male	57 (81.4%)	119 (75.8%)	15 (83.3%)	
- Female	13 (18.6%)	38 (24.2%)	3 (16.7%)	
Glucose metabolism				
- At admission:				
HbA1c, % (SD)	5.1 (0.4)	5.4 (0.3)	6.5 (1.0)	**<0.01**
FPG, mmol/l (SD)	4.8 (0.4)	5.3 (0.6)	7.0 (1.5)	**<0.01**
2hPG, mmol/l (SD)	6.3 (0.9)	7.9 (2.1)	13.3 (3.3)	**<0.01**
- At follow-up:				
HbA1c, % (SD)	5.2 (0.3)	5.5 (0.4)	6.1 (0.5)	**<0.01**
FPG, mmol/l (SD)	5.0 (0.4)	5.6 (0.5)	7.1 (1.4)	**<0.01**
2hPG, mmol/l (SD)	5.1 (1.0)	6.8 (2.3)	11.9 (3.2)	**<0.01**
hs-CRP, mg/L median (IQR)	0.7 (0.5, 1.3)	1.4 (0.7, 2.7)	1.4 (0.9, 1.4)	**<0.01**
Insulin, mU/L (SD)	10.47 (6.58)	13.51 (8.90)	16.42 (11.11)	0.07
HOMA-IR, units (SD)	2.33 (1.56)	3.40 (2.41)	4.96 (3.86)	**<0.01**
TPA, mm^2^ (SD)	86.8 (57.2)	108.9 (66.1)	118.0 (70.7)	**0.04**
Grey-scale median (SD)	38 (11)	36 (10)	36 (11)	**0.69**
Mean cIMT, mm (SD)	0.87 (0.15)	0.91 (0.14)	0.90 (0.15)	0.28
Carotid plaque				0.13
- No plaque	36 (51.5%)	52 (33.1%)	5 (27.8%)	
- Significant plaque	34 (48.5%)	105 (66.9%)	13 (72.2%)	
BMI, kg/m^2^ (SD)	27.8 (4.0)	28.8 (4.1)	31.4 (4.8)	**<0.01**
WHR (SD)	0.96 (0.07)	0.98 (0.08)	1.01 (0.06)	**0.02**
Smoking status:				**0.03**
- Never	44.3%	28.0%	22.2%	
- Previous	44.3%	44.0%	44.5%	
- Current	11.4%	28.0%	33.3%	
FH of CAD^a^	67.1%	64.3%	72.2%	0.78
Hypercholesterolemia	48.6%	43.9%	55.6%	0.54
Hypertension	52.9%	57.3%	66.7%	0.60
Previous CAD	35.7%	30.6%	50.0%	0.22
SBP, mmHg (SD)	139 (22)	139 (23)	137 (18)	0.93
DBP, mmHg (SD)	77 (12)	78 (13)	75 (12)	0.71
ACS				0.57
- UAP	31.4%	28.7%	38.9%	
- NSTEMI	45.7%	38.9%	33.3%	
- STEMI	22.9%	32.4%	27.8%	
CAD^b^				**0.03**
- 0-vessel	10.8%	7.1%	0%	
- 1-vessel	50.0%	35.4%	33.3%	
- 2-vessel	19.6%	37.0%	27.8%	
- 3-vessel	19.6%	20.5%	38.9%	
Gensini score (SD)	38.2 (37.5)	39.1 (32.4)	60.0 (36.3)	**0.04**

NGM: normal glucose metabolism, T2DM: type 2 diabetes mellitus, SD: standard deviation, FPG: fasting plasma glucose, 2hPG: 2-hour plasma glucose, hs-CRP: high-sensitivity C-reactive protein, IQR: interquartile range, HOMA-IR: homeostatic model assessment insulin resistance, TPA: total plaque area, cIMT: carotid intima-media thickness, BMI: body mass index, WHR: waist to hip ratio, FH: family history, CAD: coronary artery disease, SBP: systolic blood pressure, DBP: diastolic blood pressure, ACS: acute coronary syndrome, UAP: unstable angina pectoris, NSTEMI: non-ST elevation myocardial infarct, STEMI: ST elevation myocardial infarct

^a^CAD with 1^st^ degree family member

^b^≥70% of lumen reduction

**Table 3:** Carotid plaque burden among patients with IFG, IGT and combination of IFG and IGT.

	IFG (n = 50)	IGT (n = 42)	IFG & IGT (n = 52)	p-value
TPA, mm^2^ (SD)	87.8 (53.1)	127.4 (70.4)	123.1 (70.9)	<0.01
Mean cIMT, mm (SD)	0.88 (0.16)	0.91 (0.13)	0.92 (0.13)	0.30
Carotid plaque				0.67
- No plaque	36.0%	28.6%	28.8%	
- Significant plaque	64.0%	71.4%	71.2%	

IFG: impaired fasting glucose, IGT: impaired glucose tolerance, SD: standard deviation, TPA: total plaque area, cIMT: carotid intima-media thickness.

When the relationship between the different glucometabolic assessments and carotid plaque were examined, only the 2hPG during admission and at follow-up showed statistically significant unadjusted OR of having significant plaque in the carotid arteries, being 2.37 (95% CI 1.36–4.18) and 2.25 (1.21–4.35), respectively. For HbA1c and FPG classification of dysglycemia the unadjusted OR for the detection of atherosclerotic plaque in the carotids did not reach statistical significance (Table 4A).

**Table 4:** Carotid plaque burden by different glucose methods.

a) The OR of having significant plaque in the carotid arteries of patients diagnosed with dysglycemia based on different glucometabolic assessments compared to normal glucose level.				
	Unadjusted OR, (95% CI)	p-value	^a^Adjusted OR, (95% CI)	p-value
HbA1c				
- Dysglycemia during admission	1.31 (0.69–2.57)	0.41	0.89 (0.44–1.93)	0.74
- Dysglycemia at follow-up	1.74 (0.96–3.27)	0.07	1.58 (0.82–3.13)	0.18
FPG				
- Dysglycemia during admission	1.46 (0.80–2.76)	0.23	1.52 (0.77–3.08)	0.24
- Dysglycemia at follow-up	1.44 (0.85–2.46)	0.18	1.61 (0.89–2.96)	0.12
2hPG				
- Dysglycemia during admission	**2.37 (1.36–4.18)**	**<0.01**	**1.92 (1.05–3.55)**	**0.04**
- Dysglycemia at follow-up	**2.25 (1.21–4.35)**	**0.01**	1.51 (0.74–3.18)	0.26
b) Difference in TPA among patients diagnosed with dysglycemia based on different glucometabolic assessment compared to normal glucose level.				
	Unadjusted Estimate (SE)	p-value	^a^Adjusted Estimate (SE)	p-value
HbA1c				
- Dysglycemia during admission	**27.3 (10.3)**	**<0.01**	15.1 (9.4)	0.11
- Dysglycemia at follow-up	14.5 (9.4)	0.12	13.2 (8.5)	0.12
FPG				
- Dysglycemia during admission	6.3 (9.8)	0.52	7.3 (8.9)	0.41
- Dysglycemia at follow-up	1.67 (8.5)	0.84	6.9 (7.8)	0.38
2hPG				
- Dysglycemia during admission	**35.0 (8.3)**	**<0.01**	**25.5 (7.6)**	**<0.01**
- Dysglycemia at follow-up	**37.0 (9.2)**	**<0.01**	**24.0 (8.8)**	**<0.01**

OR: odds-ratio, CI: confidence interval, SE: standard error, 2hPG: 2-hour plasma glucose, FPG: fasting plasma glucose.

^a^Adjusted for age, gender, hypertension, hypercholesterolemia, smoking status, and BMI

When adjusted for conventional atherosclerotic risk factors, the OR for patients diagnosed with dysglycemia according to the 2hPG during admission of having significant plaque in the carotid arteries was 1.92 (95% CI 1.05–3.55) and 1.51 (0.74–3.18) at follow-up. However, the ORs for HbA1c and FPG remained statistically non significant.

The relationship between the diagnosis of dysglycemia by the three different methods and TPA is shown in Table 4B. Only 2hPG showed a statistically significant association with TPA both during admission and follow up. HbA1c during admission was associated with an increase in mean TPA but was not statistically significant at the follow up. Based on the multiple regression model, after adjusting for conventional atherosclerotic risk factors, TPA was significantly associated with 2hPG during admission and at follow-up with 25.5 mm^2^ (SE 7.6, p<0.01) and 24.0 mm^2^ (SE 8.8, p<0.01) greater TPA, respectively, compared to those with normal glucose level.

The unadjusted OR of patients diagnosed with prediabetes, T2DM and dysglycemia of having significant atherosclerotic plaque in their carotid arteries compared to patients with NGM was 2.14 (95% CI; 1.21–3.81), 2.75 (95% CI; 0.93–9.34) and 2.19 (95% CI; 1.25–3.87), respectively (Table 5A, model 1).

**Table 5:** Glucose metabolic derangements and carotid plaque burden.

a) The OR of having significant plaque in the carotid arteries among patients diagnosed with prediabetes, T2DM or dysglycemia defined as any of abnormal 2hPG, HbA1C and fasting glucose.						
	Model 1		Model 2		Model 3	
	Unadjusted OR (95% CI)	p-value	^a^Adjusted OR (95% CI)	p-value	^b^Adjusted OR (95% CI)	p-value
Prediabetes	**2.14 (1.21–3.81)**	**0.01**	**2.14 (1.13–4.12)**	**0.02**	1.78 (0.83–3.88)	0.14
T2DM	2.75 (0.93–9.34)	0.08	2.50 (0.75–9.51)	0.15	2.18 (0.44–12.03)	0.35
Dysglycemia	**2.19 (1.25–3.87)**	**<0.01**	**2.17 (1.15–4.15)**	**0.02**	1.77 (0.83–3.84)	0.14
b) Difference in TPA among patients diagnosed with prediabetes, T2DM and dysglycemia defined as any of abnormal 2hPG, HbA1C and fasting glucose.						
	Model 1		Model 2		Model 3	
	Unadjusted Estimate mm^2^ (SE)	p-value	^a^Adjusted Estimate mm^2^ (SE)	p-value	^b^Adjusted Estimate mm^2^ (SE)	p-value
Prediabetes	**22.1 (9.3)**	**0.02**	**22.5 (8.5)**	**<0.01**	9.6 (9.9)	0.34
T2DM	31.2 (17.0)	0.07	29.4 (15.5)	0.06	1.1 (19.5)	0.95
Dysglycemia	**23.0 (9.2)**	**0.01**	**23.1 (8.4)**	**<0.01**	10.0 (10.0)	0.32

OR: odds ratio, CI: confidence interval, SE: standard deviation, T2DM: type 2 diabetes mellitus

^a^Adjusted for age, gender, hypertension, hypercholesterolemia, smoking status, and BMI

^b^ Adjusted for age, gender, hypertension, hypercholesterolemia, smoking status, BMI and 2hPG during admission and at follow-up

From the multiple logistic regression analysis after adjusting for conventional atherosclerotic risk factors the ORs of having significant atherosclerotic plaque for patients diagnosed with prediabetes, T2DM and dysglycemia (determined by abnormal results in either 2hPG, HbA1C or FPG) compared to NGM was 2.14 (95% CI 1.13–4.12), 2.50 (0.75–9.51) and 2.17 (95% CI 1.15–4.15), respectively (Table 5A, model 2). To examine if any of the dysglycemic metabolic determinants might explain this association we additionally adjusted for 2hPG, HbA1C and fasting glucose both during admission and at follow-up. When adjusted for 2hPG the statistical significance of the ORs for patients diagnosed with prediabetes and dysglycemia of having significant atherosclerotic plaque compared to NGM was lost and became 1.78 (95% CI 0.83–3.88) and 1.77 (95% CI 0.83–3.84), respectively (Table 5A, model 3). When adjusted for FPG during admission and at follow-up the ORs were also attenuated 1.94 (95% CI 0.89–4.29) and 1.93 (95% CI 0.89–4.27) for prediabetes and dysglycemia respectively, but remained significant when adjusted for HbA1c during admission and at follow-up.

Based on regression analysis, the diagnosis of prediabetes, T2DM and dysglycemia was associated with an increase in TPA of 22.1 mm^2^ (SE: 9.3, p = 0.02), 31.2 mm^2^ (SE: 17.0, p = 0.07) and 23.0 mm^2^ (SE 9.2, p = 0.01), respectively, compared to patients with NGM (Table 5B, model 1). From the multiple variable regression model after adjusting for conventional atherosclerotic risk factors a significant increase in TPA was associated with the diagnosis of prediabetes and dysglycemia (Table 5B, model 2). When additionally adjusted for 2hPG during admission the difference in TPA among patients with prediabetes and dysglycemia was attenuated to 9.6 mm^2^ (SE: 10.0, p = 0.34) and 10.0 mm^2^ (SE 10.0, p = 0.32), respectively, compared to patients with NGM (Table 5B, model 3) which is a reduction of the effect sizes by 57.3% and 56.7% for prediabetes and dysglycemia, respectively. When adjusted independently for FPG and HbA1c during admission and at follow-up the difference in TPA among patients diagnosed with prediabetes and dysglycemia still remained statistically significant.

## Discussion

In the current study, a high prevalence of atherosclerotic plaque in the carotid arteries was detected with incremental increase in TPA in ACS patients diagnosed with prediabetes and T2DM compared to patients with NGM. Detection of dysglycemia by measurement of 2hPG rather than FPG or HbA1c identified increased risk of significant atherosclerotic plaque and increased TPA. Therefore, newly detected glucose derangements are associated with increased atherosclerotic burden in ACS patients.

All patients in the present study had established atherosclerotic disease in their coronary arteries. Therefore it was expected to detect a high prevalence of atherosclerotic plaque in the carotids in the study population. In this study the prevalence for significant atherosclerotic plaque was 62% compared to 10% reported from a general population study in Iceland that used the same ultrasound protocol as in the current study [16]. When compared to an age- and gender-matched control group from that population study the prevalence of significant plaque was 20% and mean TPA 39.1 mm^2^ compared to 103.3 mm^2^ in the present study. This highlights the high atherosclerotic burden found in patients with CVD compared to the general population.

When the individual glucose measurement methods were evaluated separately the 2hPG for dysglycemia during admission and at follow-up were found to be independent predictors of having significant atherosclerotic plaque in the carotid arteries. Likewise, patients diagnosed with dysglycemia on the 2hPG had significantly increased TPA compared to patients with NGM. The 2hPG identifies patients who are nearly or completely insulin resistant, a condition where the liver and muscles require more insulin to suppress the hepatic glucose production and glucose uptake, respectively, that results in postprandial hyperglycemia [19, 20]. While the pancreatic β-cells are able to increase their insulin production and secretion, causing hyperinsulinemia, the glucose tolerance remains normal. In time, however, the postprandial glucose levels and subsequently the FPG levels rises due to increased insulin resistance and failure of the β-cells to produce insulin, leading to overt T2DM [21].

Prediabetes and dysglycemia were found to be independent predictors of having significant atherosclerotic plaque in the carotid arteries. A significant association was also detected between TPA and metabolic derangements whether defined as prediabetes or dysglycemia. No significant association was found between T2DM and TPA but there was a clear trend in that direction. After being adjusted for conventional CVD risk factors the association between dysglycemia and TPA remained significant. However, when additionally adjusting for different glucose metabolic measurements the 2hPG had a greater effect on the relationship between dysglycemia and TPA as well as the OR of having significant plaque compared to FPG and HbA1c. This indicates that among the three methods of glucometabolic measurement 2hPG has the strongest relation to atherosclerosis development in this group of patients with ACS.

The 2hPG, based on the OGTT, had stronger association with increased TPA and significant plaque in the carotid arteries than either FPG or HbA1c.

Elevated 2hPG is an indicator of insulin resistance and hyperinsulinemia. Insulin resistance and hyperinsulinemia have been suggested to cause vascular dysfunction and an increase in reactive oxygen species, which leads to vascular inflammation and proliferation of smooth muscle cells that, in turn, accelerate the atherosclerotic process [22]. Also, postprandial hyperglycemia represents the highest blood glucose levels during the day and the greatest fluctuations in glycaemia that could contribute to abnormal endothelial function [21]. Finally, patients with postprandial hyperglycemia have higher prevalence of metabolic syndrome, with central obesity, dyslipidemia and hypertension, which in itself increases risk for CVD [21]. Insulin resistance and hyperinsulinemia are conditions that are undetected for many years that contribute to greater atherosclerotic disease and increased risk for CVD. Our results concur with these findings in that 2hPG was found to have a stronger association with atherosclerosis compared to FPG and HbA1c.

To our knowledge, this is the first study to evaluate TPA in patients with ACS in relation to glucose metabolism classification. Increased TPA in patients newly diagnosed with prediabetes and dysglycemia suggests that these patients have had undiagnosed dysglycemia for some time before being admitted with ACS, causing accelerated atherosclerotic plaque formation and perhaps leading to plaque instability and rupture which is a key process in the development of ACS. Our results also support this assumption in that prediabetes and dysglycemia are shown to be independents risk factors of having significant plaque on carotid ultrasound examination. These findings are in line with previous studies indicating a high proportion of undiagnosed dysglycemia in the general population and they underscore the importance of timely preventive measures to enhance public health [23]. Patients who are diagnosed with dysglycemia should receive lifestyle counselling, mainly improving diet and increased physical activity to promote weight loss and lower cardiovascular risk [24]. Patients diagnosed with prediabetes and T2DM could also be offered pharmacotherapy with metformin to reduce risk of developing T2DM and reduce risk of myocardial infarction, respectively [25, 26]. In addition, it has been reported that reducing post-prandial hyperglycemia with acarbose, an alpha-glycosidase inhibitor, can attenuate the progression of IMT in patients with IGT [27].

A limitation in the present study is a low prevalence of T2DM that is due to the strict diagnostic criteria applied for T2DM and a relatively low prevalence of T2DM in the general population in Iceland compared to other western countries [28]. Due to this low prevalence the power to detect significant association between T2DM and TPA is limited.

## Conclusion

A high prevalence of atherosclerotic plaque in the carotid arteries was detected with incremental increase in TPA in ACS patients diagnosed with prediabetes and T2DM compared to patients with NGM. Patients diagnosed with dysglycemia according to the 2hPG were at increased risk of significant atherosclerotic plaque in their carotid arteries and had significantly increased TPA. The importance of the 2hPG in the risk stratification of these patients is reflected by the fact that after adjustment for conventional risk factors and the 2hPG no increased burden of atherosclerotic plaque remains, indicating the role of OGTT as a predictor of increased carotid plaque burden. Thus newly detected metabolic derangements by screening are an important indicator of accelerated atherosclerotic plaque formation in the ACS population. Timely detection and treatment of dysglycemia might prove advantageous for the future care in this high-risk population.

## Supporting information

S1 File Available dataset. (CSV)
